# Psychosocial and occupational risk perception among health care workers: a Moroccan multicenter study

**DOI:** 10.1186/s13104-015-1326-2

**Published:** 2015-09-04

**Authors:** Doina Ileana Giurgiu, Christine Jeoffrion, Benjamin Grasset, Brigitte Keriven Dessomme, Leila Moret, Yves Roquelaure, Alain Caubet, Christian Verger, Chakib El Houssine Laraqui, Pierre Lombrail, Christian Geraut, Dominique Tripodi

**Affiliations:** Department of Occupational Medicine and Environment Health, University Hospital of Nantes, 5 rue du doyen Boquien, 44093 Nantes, France; “Lucian Blaga” University of Sibiu, 10 Victoriei Boulevard, 550024 Sibiu, Romania; Psychology Laboratory of Pays de la Loire, UPRES EA 4638, Chemin La Censive du Tertre, 44312 Nantes, France; Department of Public Health, Nantes University Hospital, 35 rue Saint Jacques, 44000 Nantes, France; Laboratory of Ergonomics Epidemiology Health and Work, LEEST-UA InVS, IFR 132-UPRES EA 4336, Faculty of Medicine, University Hospital, University of Angers, 4 rue Larrey, 49933 Angers Cedex, France; Occupational Medicine Department, University Hospital of Rennes, 6 rue Henri Le Guilloux, 35000 Rennes, France; Graduate School of Health Engineering and Project Management, 24 rue Lafontaine, Quartier Racine, 20100 Casablanca, Morocco; Public Health Department, SMBH, Paris 13 University, 74 Avenue Marcel Cachin, 93017 Bobigny, France

**Keywords:** Occupational stress, Risk exposure, High strain, Public hospital, Medication use

## Abstract

**Background:**

International studies on occupational risks in public hospitals are infrequent and only few researchers have focused on psychosocial stress in Moroccan Health Care Workers (HCWs). The aim of this study was to present and analyze Moroccan HCWs occupational risk perception. Across nine public hospitals from three Moroccan regions (northern, central and southern), a 49 item French questionnaire with 4 occupational risks subscales, was distributed to 4746 HCWs. This questionnaire was based on the Job Content Questionnaire. Psychosocial job demand, job decision latitude and social support scores analysis were used to isolate high strain jobs. Occupational risks and high strain perception correlation were analyzed by univariate and multivariate logistic regression.

**Results:**

2863 HCWs (60 %) answered the questionnaire (54 % women; mean age 40 years; mean work seniority 11 years; 24 % physicians; 45 % nurses). 44 % of Moroccan HCWs were at high strain. High strain was strongly associated with two occupational categories: midwives (2.33 OR; CI 1.41–3.85), full-time employment (1.65 OR; CI 1.24–2.19), hypnotics and sedatives use (1.41 OR; CI 1.11–1.79), analgesics use (1.37 OR; CI 1.13–1.66).

**Conclusion:**

Moroccan HCWs, physicians included, perceive their job as high strain. Moroccan HCWs use of hypnotics, sedatives and analgesics is high. Risk prevention plan implementation is highly recommended.

**Electronic supplementary material:**

The online version of this article (doi:10.1186/s13104-015-1326-2) contains supplementary material, which is available to authorized users.

## Background

The workplace is an important contributor to a multitude of illnesses and is a determinant of the individual’s well-being. Among numerous risks found in the workplace, one entity has emerged lately to be a major public health concern and a challenge to the occupational health research field: the psychosocial risks (PSR). Psychosocial risks appear to be at the center of the intricate architecture of the work conditions.

Previous studies support the influence of negative organizational climate on nurses’ health [1], as well as the interactions between the physical and psychosocial risks factors that can generate musculoskeletal disorders [2]. The impact of occupational safety climate in hospitals confirms the link between PSR and workers’ health [3, 4]. In Europe, according to the working conditions surveys, the influence of PSR factors in the workplace was the second major change in working environments [5]. The European Agency for Safety and Health at Work (EU-OSHA), in its report on emerging risks [6] indicated that there is a strong correlation between variables considered for inclusion in the Occupational Safety Health composite score. An imbalance between the number of industrial accidents and the number of occupational illnesses has also been noted: even if the first is decreasing, the latter is increasing [7]. In France, indicators defined by experts permit to demonstrate that workplace PSR factors often contribute to the occurrence of health problems: cardiovascular diseases, mental health disorders or musculoskeletal disorders [8].

As the workforce in the European Union (EU) is ageing and the work intensity is constantly increasing, questions on steps to be taken to keep the workforce active have arisen, bringing out the need for a well-designed employment policy [9]. The fact that over 40 million people in the EU are suffering of consequences of work-related stress, which translates in over 20 billion € of health and absenteeism costs [10], goes to underline the economic and social importance of addressing the issue of work-related psychosocial risks.

The European Commission’s (EC) guidelines on work-related stress itemized the steps to be taken. The first step was identifying the risk, with its sources and consequences, by the means of monitoring “job content, working conditions, terms of employment, social relations at work, health, well-being and productivity” [11]. One of the important methods of detecting work-related stress, therefore PSR, is using a risk-perception evaluation, since the individual’s subjective experience will be the generator of symptoms and diseases: from transient increase in heart rate and blood pressure to developing cardiovascular diseases, and from minor indicators of anxiety or depression to clinical developments of mental health problems. EC has identified the significant proportion of employees who acknowledge the impact work has on their health, in its *Improving quality and productivity at work. Community strategy 2007*–*2012 on health and safety at work*: “35 % of workers on average feel that their job puts their health at risk” [12].

Currently, international studies on occupational risks in the public hospitals are infrequent, as are researchers concentrating on psychosocial stress in Moroccan HCWs [13–16], many focusing on ethnic groups living in other countries or on patients with different conditions. The 2008 study of Laraqui et al. [17] was one of the very few suggesting the need for occupational stress evaluation in Moroccan HCWs, based on findings of high stress prevalence, and the necessity for working conditions improvement.

The objectives of the present study were to assess psychosocial risk perception among Moroccan HCWs using a validated questionnaire and to analyse occupational risk factors’ impact in Moroccan public hospitals. Attention was given to different aspects which influence the occurrence of “high strain” situations: ergonomics, working conditions, and to the use of analgesic, hypnotic and sedative medication. The main goal was to develop a model for high strain perception that could be used for crafting and implementing a specific prevention plan.

## Methods

### Sample

The study was a cross-sectional multicenter investigation, conducted in Moroccan public hospitals of the three Moroccan regions: northern, central and southern. The northern region included towns above the line which joined the cities of Sidi-Kacem and Taza; the northern region hospitals were those of Kenitra, Oujda and Larache. The southern region included municipalities below the line which joined the cities of Safi and Beni Mellal; the southern region hospitals were those of Agadir and Marrakech. The central region (between the two described lines) or the Casablanca region covered hospitals of Baouafi, Sekkat, Settat and Khouribga. All public hospitals’ staff was targeted. Our research obtained the approval of the Nantes Regional Ethics Committee (Comité de Protection des Personnes Ouest IV) on December 7th, 2010, for research on “Work Organisation Cancer and Health” (No. DC 2010-1199). The Moroccan Public Health Ministry’s and the Moroccan Occupational Health Ethic Committee (Ref. no 312-10, Casablanca) authorization were obtained before beginning the investigation.

### Material

The occupational risks questionnaire was distributed. It had 49 questions grouped in four subscales. The first 30 items on work and psychosocial relations (decision latitude, psychological demand and social support—sum of supervisor and coworker support) were extracted from Karasek’s Job Demand Questionnaire (JCQ) [18]. The next items stemmed from a validated French questionnaire [19, 20]: eight items targeted workplace ergonomics and three items addressed workplace environment risks. The last 8 items focused on passive smoking, alcoholism and medication use (hypnotics, sedatives and analgesics) and were taken from the “Adults Health Barometer” of the French National Institute of Prevention and Health Education.

### Procedure

The questionnaire was distributed and collected upon completion by Moroccan occupational medicine students. They informed the staff on the questionnaire’s purpose, items and anonymity. All 4746 employees from the nine public hospitals were considered eligible. All the employees and the Health and Safety Committee was informed of the study and approved it.

### Data analysis

Moroccan occupational medicine students gathered the data. They used an Excel mask designed by the Medical Evaluation and Therapeutic Education Office of the Medical Information and Public Health Evaluation Department of Nantes University Hospital. Data was analyzed with ASS/STAT 8.2 and SPSS 13.0. Missing answers questionnaires were excluded from the analysis. Descriptive analysis of data was made: answer percentage for qualitative variables, mean and standard deviation for quantitative variables. Job Decision Latitude (JDL), Psychological Job Demand (PJD) and Social Support (SoSu) scores were calculated according to Karasek et al. [18] and Niedhammer and al. [21]. Threshold values for high PJD, low JDL and a low SoSu were respectively set to 24, 72 and 22 [21]. High strain occupations had PJD score over 24 and JDL score under 72. A SoSu score below 22 denoted social isolation. The DETA (French version of CAGE questionnaire for alcoholism screening) score could not be calculated due to the lack of answers to those items.

Bivariate analysis was performed with Chi squared test for categorical variables (e.g.: between medication use and drug consumption), with Student’s *t* test for between groups comparison of metric variables and with Pearson’s correlation coefficient for comparison of metric variables. Factors influencing the scores were determined by one-way ANOVA and by MANOVA for occupational categories adjustment. Step-by-step logistic regression was used for identifying factors connected to high PJD associated with low JDL.

## Results

### Descriptive data

Two thousand eight hundred and sixty-three HCWs (60 %) answered the questionnaire. 54 % of the HCWs were women. Mean age was 40.4 ± 10.2 years. Mean work seniority was 11.3 ± 9.9 years. 97 % of HCWs had a permanent employment contract and 79 % worked full-time. The participation rate varied among the different hospitals, with leading scores from Larache (northern Morocco) and Sekkat (Casablanca) hospitals (94 and 91 %) and lowest score (38 %) from Marrakech hospital (southern Morocco). The participation rate was higher in the administration departments (67 %) and lower in surgery and ancillary departments (57 %). The ancillary staff had the highest participation rate (74 %), followed by the custodial staff and the administrative staff (73 and 70 %). The technical staff (27 %), the psychologists (33 %) and the nursing aides (37 %) had the lowest participation in the study (Table 1).

**Table 1:** Participation rate by public hospital location, type of department and occupational category

	HCWs (*n*)	Respondents (*n*)	Participation rate (%)
Public Hospital			
Baoufi Casablanca	308	181	59
Sekkat Casablanca	274	248	91
Settat	336	234	70
Khourigba	480	346	72
Agadir	888	562	63
Marrakech	1046	400	38
Kénitra	453	258	57
Oujda	650	343	53
Larache	311	291	94
Department			
Internal Medicine	983	589	60
Administration	646	435	67
Ancillary	733	418	57
Surgery	675	382	57
OBG	529	327	62
Paediatrics	241	156	65
OR-ICU-ER	939	556	59
Occupational category			
Nurse	1340	788	59
Physician	1013	679	67
Specialist nurse	838	511	61
Administrative	483	337	70
Midwife	226	156	69
Ancillary staff	132	97	74
Other	251	69	28
Custodial staff	84	61	73
Nursing aide	129	48	37
Rehabilitation medicine staff	62	39	63
Nursing management	44	30	68
Technical staff	109	29	27
Social worker	29	17	59
Psychologist	6	2	33
Total	4746	2863	60

*OBG* obstetrics and gynecology, *OR* operating room, *ICU* intensive care unit, *ER* emergency room

Internal consistency of the study was determined for each subscale; Cronbach’s alpha coefficients were 0.596 for JDL, 0.383 PJD, 0.764 for SoSu, 0.737 for workplace ergonomics, 0.45 for environmental risks and 0.516 for medication use. SoSu and PJD scores were weakly correlated (r = −0.025, NS). JDL and PJD scores were correlated (r = 0.110, p < 0.01), as well as JDL and SoSu scores (r = 0.248; p < 0.01).

### Psychosocial risks

JDL, PJD and SoSu scores were diverse among occupational categories (p < 0.001) (Table 2).

**Table 2:** Answer influencing factor: occupational category

Decision latitude				Psychological demand				Social support			
Occupational category	*n*	Mean	SD	Occupational category	*n*	Mean	SD	Occupational category	*n*	Mean	SD
Technical staff	25	53.9	10.4	Psychologist	2	23.0	0	Technical staff	27	19.9	4.1
Other	58	62.1	9.3	Rehabilitation medicine staff	39	23.1	3.2	Midwife	145	20.9	3.3
Administrative	297	62.9	9.9	Nursing aide	39	23.3	2.7	Other	48	21.3	3.4
Social worker	13	62.9	10.4	Administrative	299	23.6	3.4	Nursing management	27	21.5	3.9
Custodial staff	60	64.3	13.8	Social worker	16	24.0	3.5	Nurse	716	21.6	3.6
Nursing aide	41	64.4	6.8	Other	53	24.1	2.7	Physician	646	21.6	3.8
Ancillary staff	88	65.1	10.6	Technical staff	27	24.2	2.8	Specialist nurse	469	22.0	3.7
Nursing management	27	65.4	7.8	Specialist nurse	463	24.2	3.3	Nursing aide	43	22.0	3.6
Midwife	142	65.6	9.9	Ancillary staff	88	24.4	3.4	Rehabilitation medicine staff	38	22.0	3.3
Nurse	729	66.2	9.4	Custodial staff	60	24.6	2.5	Ancillary staff	95	22.1	3.7
Specialist nurse	461	66.9	8.8	Nurse	717	24.7	3.2	Administrative	302	22.3	3.8
Physician	630	69.5	9.0	Physician	630	25.0	3.3	Social worker	12	22.6	3.7
Rehabilitation medicine staff	38	74.5	9.9	Nursing management	26	25.2	2.8	Custodial staff	60	23.4	5.4
Psychologist	2	83.0	9.9	Midwife	148	25.8	3.1	Psychologist	2	26.5	2.1
Total	2611	66.5	9.8		2607	24.6	3.3		2630	21.8	3.8

JDL, PJD and SoSu were independent of gender. JDL and SoSu decreased as the age of respondents increased (JDL correlation coefficient = −0.078, p < 0.01; SoSu correlation coefficient = −0.075, p < 0.01), situation persistent after adjustment for occupational category. The same JDL and SoSu pattern was found related to work seniority, but after adjustment for occupational category the situation persisted only for JDL. PJD was independent of age and seniority. JDL and PJD were independent of respondents’ employment type and contract. Temporary employees had higher social support (p < 0.001). Full-time employees scored lower in JDL and SoSu (p < 0.001), but higher in PJD (p < 0.001).

### Other risks: use of hypnotics or sedatives, use of analgesics, working conditions

20 % of respondents reported taking hypnotics or sedatives on a regular basis (more than once during the week preceding the investigation), with an equal proportion of men and women. On average they were older (43.2 ± 9.9 years versus 40.3 ± 10.0 years, p < 0.001). 41 % of respondents reported taking analgesics regularly (more than once during the week preceding the investigation). Most of them were women (43 % women versus 39 % men, p < 0.05) and were older (41.9 ± 10.1 years versus 40.3 ± 9.9 years, p < 0.001). Hypnotics or sedatives use significantly correlated to low JDL (p < 0.001). It also correlated to high PJD (p < 0.05) and lower SoSu (p < 0.01). Use of analgesics also correlated significantly to low JDL (p < 0.05), as well as to high PJD (p < 0.001) and lower SoSu (p < 0.05). The questions regarding the alcohol intake could not be used because the survey was accidentally carried out in the month of Ramadan (when, following a religious principle, use of alcohol is prohibited).

### High strain

**Fig. 1:**
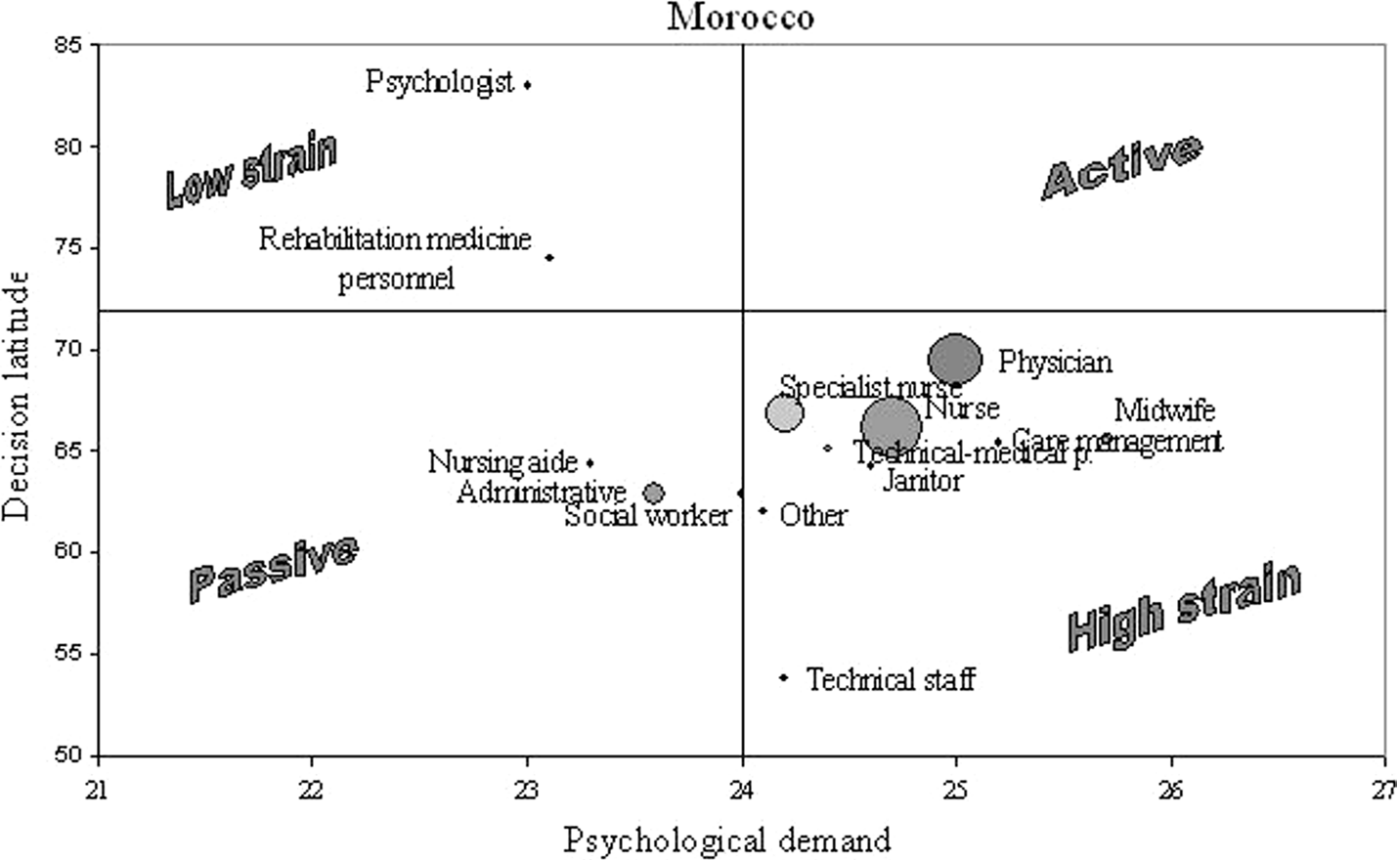
Psychological job demand (PJD) and job decision latitude (JDL) among Moroccan Health Care Workers. Threshold values for high PJD, low JDL were respectively set to 24, 72. High strain occupations had PJD score over 24 and JDL score under 72

The respondents with the highest risk (high strain: occupational categories with the riskier combination of low job decision latitude, high psychological job demand and low social support) were midwives and nursing management. High strain occupations also included nurses, physicians and ancillary staff. No occupational category placed itself in the active job quadrant—high demand and high control (Fig. 1).

High strain was associated with employment contract and type, occupational category, workplace environment (noise level, lighting) and use of hypnotics and sedatives (p < 0.001) (Table 3).

**Table 3:** High strain and occupational risk factors in Moroccan HCWs: multivariate analysis

Problematic context: high strain	*n*	%	*p*	Crude OR	Crude CI	Adjusted OR	CI
Group, *n* = 2472							
Casablanca region	387	50	***	1		1	
Northern Morocco	399	51		0.62	0.50–0.75	1.03	0.82–1.31
Southern Morocco	353	38		1.05	0.86–1.29	0.73	0.56–0.96
Gender, *n* = 2453							
Female	620	48	*	1		1	
Male	512	45		0.89	0.76–1.05	1.26	1.04–1.53
Age, *n* = 2440	–	–		–	–	0.99	0.98–0.99
Employment contract, *n* = 2462							
Permanent	1096	46	***	1		–	–
Temporary	37	48		1.09	0.67–1.75	–	–
Employment type, *n* = 2448							
Full-time	923	49	***	1.68	1.37–2.05	1.65	1.24–2.19
Part-time	201	36		1		1	
Occupational category, *n* = 2452							
Administrative	123	45	***	1		1	
Nursing management	16	62		1.94	0.80–4.79	2.10	0.85–5.23
Specialist nurse	181	42		0.87	0.63–1.20	0.79	0.56–1.12
Nurse	335	49		1.15	0.86–1.53	1.07	0.77–1.48
Nursing aide	16	46		1.02	0.48–2.18	0.96	0.42–2.21
Custodial staff	27	45		0.99	0.54–1.80	0.95	0.51–1.78
Midwife	94	69		2.71	1.72–4.29	2.33	1.41–3.85
Rehabilitation medicine staff	7	18		0.27	0.11–0.68	0.24	0.09–0.62
Social worker	6	50		1.21	0.34–4.37	1.34	0.36–5.08
Physician	244	41		0.84	0.62–1.13	0.65	0.47–0.91
Ancillary staff	45	54		1.43	0.85–2.42	1.33	0.75–2.34
Technical staff	12	50		1.21	0.49–3.00	0.44	0.15–1.31
Other	21	45		0.98	0.50–1.90	1.12	0.52–2.42
Accessibility in the workplace, *n* = 2504							
Satisfactory	750	42	***	1		1	
Unsatisfactory	363	52		1.50	1.26–1.78	1.24	1.01–1.53
Comfortable work posture, *n* = 2799							
Satisfactory	379	41	**	1		–	–
Unsatisfactory	698	47		1.30	1.10–1.53	–	–
Work plan height, *n* = 2799							
Satisfactory	417	41	***	1		–	–
Unsatisfactory	587	47		1		–	–
Manual handling, *n* = 2799							
Easy	489	43	**	1.31	1.11–1.55	–	–
Difficult	495	49		1		–	–
Noise level, *n* = 2799							
Correct	563	39	***	1.27	1.07–1.51	1	
Incorrect	556	51		1		1.31	1.08–1.60
Lighting or brightness level, *n* = 2799							
Correct	501	38	***	1		1	
Incorrect	624	52		1.76	1.50–2.06	1.45	1.19–1.76
Ambient temperature level, *n* = 2799							
Acceptable	409	40	***	1		–	–
Unacceptable	721	48		1.38	1.17–1.62	–	–
Exposure to hazardous chemicals, *n* = 2799							
Satisfactory	279	47	*	1		–	–
Unsatisfactory	792	46		0.96	0.79–1.16	–	–
Exposure to radiation, *n* = 2799							
Satisfactory	185	43	*	1		–	–
Unsatisfactory	881	47		1.17	0.94–1.45	–	–
Safety instructions, *n* = 2799							
Present or available	287	40	**	1		–	–
Absent or unavailable	777	47		1.29	1.08–1.54	–	–
Use of hypnotics or sedatives, *n* = 2835							
Yes	250	53	***	1.50	1.22–1.85	1.41	1.11–1.79
No	811	43		1		1	
Use of analgesics, *n* = 2835							
Yes	499	50	***	1.36	1.15–1.61	1.37	1.13–1.66
No	587	42		1		1	

*CI* confidence interval, *OR* odds ratio, *p* *p* value for Chi square test

*** *p* < 0.001, ** 0.001 < *p* < 0.01, * 0.01 < *p* < 0.05

Considering the risk factors in our model, stress level can be quantified by an equation: HS = a × PJD + b × JDL + c × SoSu + α (employment contract) + β (employment type) + γ (occupational category) + δ (work environment) + λ (care equipment availability), where HS = high strain, PJD = psychological job demand, JDL = job decision latitude, SoSu = social support.

## Discussion

This is the first cross-sectional multicenter study to assess PSR perception and to estimate occupational stress in Moroccan public hospitals. It showed a good participation rate. We believe that hospital HCWs’ very strong desire to participate in the survey (94 % participation rate in Sekkat Casablanca and 91 % participation rate in Larache) speaks in itself about awareness and need for change in working conditions. There were not many studies on Moroccan HCWs’ PSR perception, aside of the work of Laraqui et al. [17], who first focused on national, multicenter PSR evaluation in Moroccan hospitals, and found a 21.7 % prevalence of stress. Karasek’s model of PSR assessment differentiated three work dimensions: psychological demands, decision latitude and social support. By placing the worker at the core of the working systems, it has found high international audience. Though Karasek’s JCQ was translated, tested and used in many different countries [21–24], most of the investigations included one or two occupational categories. Previous studies in healthcare focused mainly on nurses, probably because they were a more homogenous and easier to access group, as opposed to the more sensitive group of physicians. Due to its specific high workload and responsibilities, the latter group is a complex target. There are even studies who excluded physicians because of the difficulty in obtaining response [24]. As Robert Karasek has already stated in his 1998 study on psychosocial job assessment through JCQ [18], one of the problems with demanding job holders was their reluctance to participate in research projects, which resulted in underreporting and scientific substantiation weakening in the cause-effect correlation. Having that in mind and the 69 % of high-strain (low JDL, high PJD and low SoSu) HCWs in our study, a 60 % participation rate in the health care sector and a 67 % rate of responsiveness in physicians are dependable data for analysis, as the second of our investigation strengths is the size and diversity of the HCWs’ group.

Significantly more Moroccan HCWs have high strain jobs compared to French university hospital HCWs [19]. Whereas the French study found many active jobs: physician, nursing management and midwife, the Moroccan study brought to attention a crowded high-strain quadrant in the JDL-PJD diagram (Fig. 1). This included all of the above occupations, alongside nurses and even custodial staff, which were passive category in the French survey. In reverse, none of the Moroccan HCW jobs qualified as active. There might be a cultural mark involved, but the main reasons were economic and organizational: Moroccan physician and nurse staffing was insufficient, the doctors’ activity was always intense and had great time pressure, the staff was poorly paid and had low degree of job independence, many HCWs had to have a second job because of low pay, the technical equipment was often inadequate, outdated or faulty. Among the main perceived occupational risks, HCWs indicated also aggression, additional workload, musculoskeletal disorders and stress. Though physician and nurse were considered active jobs [18], there are many investigations with opposite results. Columbian nurses had high demands but also high control [25]. The 2004 French report [26] on public hospital HCWs showed great level of job autonomy in physicians, but a much lower one on nurses and nursing aides. The 2011 Italian study on risk factors in health care professionals [27] showed low JDL and SoSu scores for ancillary workers, but no significant variation in PJD, which is consistent with our findings. But differences in job demands are not unusual between countries and thus cultures. Pisanti et al. [28] brought evidence of higher pressure in Italian nurses as opposed to Dutch nurses, also consistent with the difference between PJD scores in our Moroccan nurses. But there are many elements to influence job perception and thus job satisfaction: the difficult climate of economic crisis leaves its mark on job security, even if all three scores of PJD, JDL and SoSu are high enough to place the nurse in the active jobs category [24].

High strain in nurses is also not uncommon and the reasons outlined by researchers reside as well in gender disparity: women report lower levels of decision latitude [18]. The 2008 study on Moroccan HCW’s found stress to be prevalent in female workers and in paramedics [17]. We did not find any gender influence on high-strain scores. However there are grounds for high strain in women: cultural elements, tradition, or increased workload (based on unclear competencies) forced on nurses [29]. Moroccan women have a different social status, since Moroccan traditional family structure gives them the household responsibilities, which may increase their general stress level [30]. Social status and high or low level of education can also influence understanding of any questionnaire content and purpose and may affect answer, bringing in bias [31].

Another study purpose was to build a ground basis for organizational and work environment changes, to decrease occupational stress and increase job control and social support. It has already been shown that, among many consequences, high JDL and SoSu enhanced safety participation [32]. After an intervention plan would be implemented in all Moroccan public hospitals, an evaluative re-analysis of PSR would be not only necessary but of high utility [33].

Our study showed no positive correlation between age and high strain (0.99 adjusted OR, CI: 0.98–0.99), similar to results from larger and more heterogeneous groups [18], though JDL and SoSu were decreasing with age, which is similar to the French study results [20]. We must consider the risk of cardiovascular disease (CVD) that comes with age, one which is already linked to job strain [34]. In a meta-analysis of fourteen prospective cohort studies, Kivimäki et al. suggested an average of 50 % excess risk of coronary heart disease when occupational stress was present [35]. A connection between occupational stress and inflammation appears to exist, the latter triggering CVDs [36].

Since we found no explanation for temporary HCWs having higher SoSu and for senior workers having lower SoSu, there is need for further investigation. Full-time employees experienced more stress than part-time employees, which was consistent with findings from previous studies [19, 20]. There was certainly more pressure and the perceived responsibility increases in full-time contract. Longer working time enhanced work load and stress, as its imprints on scores proved: lower JDL (65.5 ± 9.6), lower SS (21.6 ± 3.9) and higher PJD (24.7 ± 3.3).

Moroccan HCWs took significantly more hypnotics and sedatives (20 %) and analgesics (40 %) than French HCWs (9 and 25 %) [20]. The use of medication was significantly associated to high strain (p < 0.001). Medication use in Moroccan HCWs proved to be much higher than other reports from hospital environments. Virtanen et al. conducted a study in 21 Finnish hospitals and found lower usage rate: 5 % for anxiolytic and hypnotic medication and 16 % for pain killers [37]. Use of sedatives and hypnotics was linked to alternative shifts, night work, stress, workload and fatigue. Analgesics use associated with musculoskeletal disorders. Alcohol intake in Moroccan HCWs could not be analyzed because of the alcohol prohibition of Ramadan month. This was not taken into account when the study was designed and led to a study limitation.

Though PJD was lower in Moroccan HCWs, it still held a high score, and when associated to low JDL it concentrated a higher percentage of high strain HCWs (44 %) than the ones reported in other studies: 16.5 % in Taiwanese HCWs [23], 25 % in European workers [38]. This raises high concern and we feel it calls for an urgent intervention. As for the different occupational categories, it must be noted that Moroccan physicians were at a higher level of strain than physicians from any other country. In our study they were not an active occupation (Fig. 1). Work overload, insufficient staffing, poor doctor-patient relationship, low hospital budget are all triggers of occupation status deterioration. All studies on perceived stress focusing on or including doctors have placed them in the high demand-high control job category. This indicates higher risk degree in Moroccan doctors for developing CVDs and mental disorders. Higher depression rates compared to general population have been reported in physicians with high work demands [39]. At the other end, Moroccan rehabilitation medicine staff was classified as having a passive job, similar to findings in other reports [39, 40], which attested a much lower degree of independence.

### Limitations and future research

Our study has encountered a number of limitations. The weak points of self-reporting must be first restated. There is no solution to avoid or limit individual variation in PSR perception. The target is a personal, subjective sensation and understanding and not an objective quantification. But there is sufficient evidence that perception is at the origin of changes in well-being and of ill health, and prevention of health alterations is occupational medicine’s ultimate goal. Participants in the study have not been randomly selected. Reasons for unresponsiveness were not requested. Results may have been influenced by exclusion of subjects due to missing item answers. Karasek’s demand-control model leaves out the effort-reward balance and the patient-caregiver relationship, and there is no data on absenteeism, presenteeism and patient admission rates, factors to be taken into account in future investigation. The questionnaire version used in Morocco wasn’t perfectly identical to the one used in the French studies (see Additional file 1) [19, 20], because some workstation ergonomics items weren’t identical and therefore not mentioned. We don’t know whether job musculoskeletal injury with concomitant sedative/hypnotic and/or analgesic use preceded high strain or if high strain caused those things. However, the study provides an accurate picture of PSR in Moroccan public hospitals. It brings forward a map of stressful occupations and it points out priorities for action. Future analysis could highlight more factors to influence occupational stress and improve our mathematical formula.

## Conclusions

The aim of this study was to carry out a multicenter investigation focused on assessing PSR perception and occupational risk in Moroccan public hospitals. Our results show that Moroccan HCWs, physicians included, have high strain activity. There were no active occupations found after PSR analysis, which show HCW’s low level of work control. Use of hypnotics, sedatives and analgesics is at high level in Moroccan HCWs and correlates with high strain. Since sedative use is high, it would be important to know whether the analgesic use is primarily NSAIDs/Tylenol vs opioids, then this is future investigation that should be done. Further research with an enlarged study pool and a more exhaustive analysis would bring more information on PSR and HCWs’ health and would help design better occupational safety and health policy, aimed at enhancing and consolidating HCWs well-being at work.

## Additional files

Additional file 1. French version of the questionnaire
