# Supplementary material for: Psychosocial and occupational risk perception among health care workers: a Moroccan multicenter study
Source: BMC Res Notes. 2015 Sep 4;8:408. doi: 10.1186/s13104-015-1326-2 (PMC4559322; doi:10.1186/s13104-015-1326-2)
Supplement: Additional file 1. — French version of the questionnaire [file 13104_2015_1326_MOESM1_ESM.doc]

# Questionnaire de Karasek, version française modifiée 07/01/2005:

**Les questions ci-dessous concernent votre travail et les relations avec votre entourage professionnel**

## Cocher une seule case par question

Fortement en d’accord tout à fait

en désaccord désaccord d’accord

1- Mon travail nécessite que j’apprenne des choses nouvelles ^1^ ^2^ ^3^ ^4^ 1 

2- Mon travail nécessite un niveau élevé de qualifications ^1^ ^2^ ^3^ ^4^ 2 

3- Dans mon travail, je dois faire preuve de créativité ^1^ ^2^ ^3^ ^4^ 3 

4- Mon travail consiste à refaire toujours les mêmes choses ^1^ ^2^ ^3^ ^4^ 4 

7- Au travail, j’ai l’opportunité de faire plusieurs choses

différentes ^1^ ^2^ ^3^ ^4^ 7 

9- Au travail, j’ai la possibilité de développer mes habiletés

personnelles ^1^ ^2^ ^3^ ^4^ 9 

6- Mon travail me permet de prendre des décisions de façon

autonome ^1^ ^2^ ^3^ ^4^ 6 

5- J’ai la liberté de décider comment je fais mon travail ^1^ ^2^ ^3^ ^4^ 5 

8- J’ai passablement d’influence sur la façon dont les choses

se passent à mon travail ^1^ ^2^ ^3^ ^4^ 8 

10- Mon travail exige d’aller très vite ^1^ ^2^ ^3^ ^4^ 10 

11- Mon travail exige de travailler très fort mentalement ^1^ ^2^ ^3^ ^4^ 11 

12- On ne me demande pas de faire une quantité excessive de

travail ^1^ ^2^ ^3^ ^4^ 12 

13- J’ai suffisamment de temps pour faire mon travail ^1^ ^2^ ^3^ ^4^ 13 

14- Je ne reçois pas de demandes contradictoires de la part

des autres ^1^ ^2^ ^3^ ^4^ 14 

15- Mon travail m’oblige à me concentrer intensément

pendant de longues périodes ^1^ ^2^ ^3^ ^4^ 15 

16- Ma tâche est souvent interrompue avant que je l’aie

terminée, je dois alors y revenir plus tard ^1^ ^2^ ^3^ ^4^ 16 

17- Mon travail est souvent mouvementé ^1^ ^2^ ^3^ ^4^ 17 

18- Je suis souvent ralenti dans mon travail parce que je dois

attendre que les autres aient terminé le leur ^1^ ^2^ ^3^ ^4^ 18 

19- Ma hiérarchie se soucie du bien-être des travailleurs qui

sont sous sa supervision ^1^ ^2^ ^3^ ^4^ 19 

20- Mon supérieur hiérarchique prête attention à ce que je dis ^1^ ^2^ ^3^ ^4^ 20 

21- Mon supérieur hiérarchique a une attitude hostile ou

conflictuelle envers moi ^1^ ^2^ ^3^ ^4^ 21 

22- Mon supérieur hiérarchique facilite la réalisation du travail ^1^ ^2^ ^3^ ^4^ 22 

23- Mon supérieur hiérarchique réussit à faire travailler les gens

ensemble ^1^ ^2^ ^3^^4^ 23 

24- Les gens avec qui je travaille sont qualifiés pour les tâches

qu’ils accomplissent ^1^ ^2^ ^3^ ^4^ 24 

25- Les gens avec qui je travaille s’intéressent personnellement

à moi ^1^ ^2^ ^3^ ^4^ 25 

26- Les gens avec qui je travaille ont des attitudes hostiles ou

conflictuelles ^1^ ^2^ ^3^ ^4^ 26 

Fortement en d’accord tout à fait

en désaccord désaccord d’accord

27- Les gens avec qui je travaille sont amicaux ^1^ ^2^ ^3^ ^4^ 27

28- Les gens avec qui je travaille s’encouragent mutuellement

à travailler ensemble ^1^ ^2^ ^3^ ^4^ 28 

29- Les gens avec qui je travaille facilitent la réalisation du travail ^1^ ^2^ ^3^ ^4^ 29 

30- Il existe des situations d’agression physique ou verbale à mon

travail  ^1^ ^2^ ^3^ ^4^ 30 

Les questions suivantes concernent l’ approche ergonomique au poste de travail et plus particulièrement l’analyse biomécanique :

(certains items peuvent ne pas s’appliquer à votre situation de travail : ne pas cocher la ligne dans ce cas)

31- L’ accessibilité à mon poste de travail est satisfaisante  ^1^ ^2^ ^3^ ^4^ 31 

32- Le confort postural à mon poste de travail est satisfaisant ^1^ ^2^ ^3^ ^4^ 32 

33- La hauteur du plan du travail est correcte  ^1^ ^2^ ^3^ ^4^ 33

34- La manutention manuelle est difficile ^1^ ^2^ ^3^ ^4^ 34 

35- La manutention de type mécanique est difficile ^1^ ^2^ ^3^ ^4^ 35 

36- La fréquence de soulèvement de charges est élevée ^1^ ^2^ ^3^ ^4^ 36 

37- La moyenne des charges que je soulève par jour  est (en Kg): ………………… 37 

38 Lors de l’exécution d’une tâche de travail, la hauteur maximale

que peut atteindre mon bras par rapport à l’épaule

(en degré par rapport à l’horizontale) est:…………degrés 38

39- Le Service où j’ exerce est suffisamment doté en

lits électriques ^1^ ^2^ ^3^ ^4^ 39 

40- Les consignes de sécurité (incendie, contrôle machine ou outil de travail…) sont présentes ou disponibles

(affichage, dossiers ou fiches à disposition par exemple): ^1^ ^2^ ^3^ ^4^ 40 

41- Je consomme regulièrement alcool ou tabac ? ^1^ ^2^ ^3^ ^4^ 41 

42- Pour me rendre au travail, j’utilise une voiture, une moto ou

un vélo  ^1^ ^2^ ^3^ ^4^ 42 

si oui, indiquez la distance : …….km

43- Je consomme régulièrement des médicaments psychotropes ^1^ ^2^ ^3^ ^4^ 43 

**Les questions suivantes concernent des risques environnementaux possibles au poste de travail:**

44- à mon poste de travail, le niveau sonore est correct ^1^ ^2^ ^3^ ^4^ 44 

45- le niveau d’éclairage ou la luminosité sont corrects ^1^ ^2^ ^3^ ^4^ 45 

46- je ne suis pas exposé à des rayonnements qui pourraient mettre en danger ma santé (RX, rayonnements

radioactifs, rayonnements électromagnétiques…) ^1^ ^2^ ^3^ ^4^ 46 

47.je ne suis pas exposé à des produits chimiques dangereux ^1^ ^2^ ^3^ ^4^ 47 

**Questions d’ordre général :**

**Pouvez vous préciser votre catégorie socio-professionnelle (cocher la case correspondante):**

Personnel administratif 

Personnel des Services de soins:

- Personnel d’encadrement 
- Infirmier spécialisés 
- Infirmier non spécialisés 
- Aide soignant 
- ASH 
- Psychologue 
- Sage femme 
- Personnel de rééducation 

Personnel éducatif ou social 

Personnel médical 

Personnel médico - technique 

Personnel technique ou ouvrier 

Statut dans l’Etablissement : Titulaire  Contractuel 

Travail à temps plein  Travail à temps partiel 

**De quel Pôle dépendez vous :**………………………

**Votre ancienneté dans l’Institution :** ……………………

**Quel est votre âge** : ………**ans;** **taille** : **cm;** **poids**: **kg;**  **sexe** **: M F**

Quelles sont selon vous les axes prioritaires d’amélioration des conditions de travail qu’il faudrait mettre en place dans votre Service  ou votre Pôle?

| **Priorité 1** |  |
| --- | --- |
| **Priorité 2** |  |
| **Priorité 3** |  |

**Nous vous remercions de votre participation active, une restitution des résultats sera communiquée à terme. L ‘analyse statistique sera conduite par le PIMESP à qui vous pouvez adresser ce questionnaire par courrier interne.**
